# Effects of Baegjinju Rice Flour Particle Size on Flour Functionality and Quality Characteristics of Batter-Coated Deep-Fat-Fried Chicken

**DOI:** 10.3390/foods15173164

**Published:** 2026-09-07

**Authors:** Dajeong Oh, Yi Ho Jeon, Youngjae Cho

**Affiliations:** 1 Department of Food Science and Technology, Pusan National University, Miryang 50463, Republic of Korea; oh9906@naver.com (D.O.); jih2792@naver.com (Y.H.J.); 2 Department of FoodTech, Pusan National University, Miryang 50463, Republic of Korea; 3 Food Tech Innovation Center, Pusan National University, Miryang 50463, Republic of Korea

**Keywords:** Baegjinju rice flour, particle size, coating performance, fried chicken, physicochemical characteristics

## Abstract

Although Baegjinju rice flour is widely used in Korea, its functionality in meat batter systems remains unclear. This study investigated the effect of Baegjinju rice flour particle size (approximately 60–177 µm) on flour physicochemical and pasting properties, rice flour gel texture, and the quality characteristics of batter-coated deep-fat-fried chicken. Rice flour was sieved into fractions and formulated into batters for chicken coating, and frying performance was assessed by measuring batter pick-up, cooking loss, moisture content, and texture with a conventional wheat flour batter included as a practical reference. As particle size decreased, water-holding capacity and heating viscosity increased, markedly increasing batter pick-up (7.54–36.68%) and reducing cooking loss (41.82–16.40%). Finer rice flour batters also improved moisture retention and reduced hardness, yielding texture comparable to that of wheat flour-coated samples. Rice flour particle size influenced hydration behavior, coating performance, and fried-product quality, supporting the use of finely fractionated Baegjinju rice flour in fried chicken batter applications.

## 1. Introduction

Deep-fat frying is widely used in the food industry because it imparts desirable texture, flavor, and appearance to foods. During frying, heat and mass transfer occur simultaneously, resulting in rapid moisture evaporation from the food matrix, crust formation, and structural changes in the coating layer [1]. In battered fried foods, the coating layer serves not only as an external covering but also as a functional barrier that influences moisture loss and textural development. Therefore, batter formulation is an important factor determining the physicochemical and structural quality of fried products [2].

Chicken breast is widely used as a raw material for fried products because of its high protein content, relatively low fat content, and neutral flavor profile. The quality of batter-coated fried chicken is strongly affected by batter consistency, adhesion to the meat surface, and structural changes occurring in the coating layer during frying. These characteristics influence coating uniformity, batter pick-up, cooking yield, moisture retention, and the texture of the final product [3]. Interest in the development of gluten-free coating systems for poultry products has continued in recent years. For example, gluten-free crispy chicken was recently developed using corn dietary fiber as a predusting material, corn flour in the liquid coating, and buckwheat flour combined with vegetable powders in the outer coating [4]. This recent study illustrates the continued research interest in cereal-based gluten-free coating formulations with appropriate viscosity, adhesion, and textural properties.

Among gluten-free coating materials, rice flour is considered a promising alternative to wheat flour because of its light color, neutral flavor, and naturally gluten-free composition. Unlike wheat flour, rice flour lacks gluten-forming proteins; therefore, its functionality in batter systems is primarily determined by starch composition, hydration properties, pasting characteristics, and viscosity [5,6]. Rice flour-based batters can form dense, starch-dominated matrices during frying and may improve coating stability and moisture retention depending on the formulation and processing conditions [7].

Rice flour particle size is an important factor determining its functional behavior. As particle size decreases, the specific surface area of rice flour particles increases, thereby altering hydration, starch gelatinization, and viscosity development. A previous study reported that reducing rice flour particle size increased hydration properties at elevated temperatures and pasting viscosity even when the effects of damaged starch were controlled [6]. Particle-size reduction during milling may also be accompanied by changes in damaged starch content. Damaged starch can increase water-binding capacity and alter the pasting and structural properties of rice flour; however, excessive damaged starch may adversely affect flour functionality and product quality [8]. In addition, amylose contributes to intermolecular associations and the formation of compact starch gel networks, thereby affecting gel strength and structural development [9]. Therefore, particle size, damaged starch content, and amylose-related functionality should be considered collectively when evaluating the functionality of rice flour in batter applications.

Baegjinju is a Korean low-amylose, semi-glutinous japonica rice cultivar [10]. Its amylose content is intermediate between that of conventional non-glutinous and glutinous rice, giving Baegjinju starch characteristics distinct from those of both rice types. Pinkaew and Naivikul compared gluten-free chicken nugget batters prepared from waxy, low-amylose, and high-amylose rice cultivars [11]. Among the unblended rice flour batters, the waxy rice batter exhibited the highest apparent viscosity, followed by the low-amylose and high-amylose rice batters. Thus, among the three cultivars examined, apparent batter viscosity tended to increase as amylose content decreased [11]. These findings suggest that the relatively high amylopectin proportion of low-amylose rice flour and its associated hydration properties may contribute to viscosity development in batter systems.

A recent comparative study confirmed the relatively low amylose content of Baegjinju and reported a high breakdown viscosity compared with the other rice cultivars examined [12]. The relatively high amylopectin proportion of Baegjinju may contribute to starch granule swelling and viscosity development, whereas the remaining amylose fraction may contribute to the formation of a structured starch network [9,12]. Based on these characteristics, the amylose composition of Baegjinju was expected to influence, at least partly, the viscosity and structural behavior of the batter. Therefore, Baegjinju was considered a suitable rice cultivar for evaluating whether particle-size refinement could enhance flour hydration and coating adhesion while maintaining favorable structural characteristics after frying. Previous studies on gluten-free coated chicken products have mainly focused on replacing wheat flour with combinations of gluten-free cereals and other coating materials or blending rice cultivars with different amylose contents [4,11]. In contrast, relatively limited information is available on the systematic fractionation of a single low-amylose rice cultivar into defined particle-size groups and the relationship between the resulting flour properties and final fried-product quality. Furthermore, previous studies on rice flour particle size and damaged starch have primarily examined bread, dumplings, and other starch-based products rather than batter-coated meat products [6,8]. Therefore, the influence of particle size within a single low-amylose rice cultivar on coating performance and fried chicken quality remains insufficiently understood.

Therefore, this study investigated the effects of Baegjinju rice flour particle size on the physicochemical and pasting properties of the rice flour fractions, the textural properties of the resulting rice flour gels, and the quality characteristics of batter-coated deep-fat-fried chicken. The rice flour fractions were evaluated for water-holding capacity, amylose content, damaged starch content, pasting behavior, and gel texture. Fried chicken quality was subsequently assessed by measuring batter pick-up, cooking loss, moisture content, and texture profile characteristics, with a conventional wheat flour batter included as a practical reference.

## 2. Materials and Methods

### 2.1. Materials

Baegjinju rice produced in Gyeonggi, Republic of Korea, was obtained from a local retailer. Commercial wheat flour (Beksul^®^ soft flour; CJ CheilJedang, Yangsan, Republic of Korea) was purchased locally and used as a conventional fried chicken batter reference. Chicken breast (Harim Co., Ltd., Iksan, Republic of Korea), sodium chloride (Beksul^®^ flower salt; CJ CheilJedang Corp., Seoul, Republic of Korea), and food-grade sodium bicarbonate (Namyang Foods, Busan, Republic of Korea) were purchased from local retailers. Commercial wheat flour was included as a practical reference representing a conventional fried chicken batter. Flour-level physicochemical analyses were restricted to the Baegjinju rice flour fractions to isolate the effect of rice flour particle size, whereas comparisons with wheat flour were conducted at the final fried-product stage. The Starch Damage Assay Kit was supplied by Megazyme International Ireland, Ltd. (Wicklow, Ireland). All chemicals and reagents were of analytical grade.

### 2.2. Preparation of Rice Flour

Rice flour was prepared by dry milling Baegjinju rice in a blade mill (HKB-100, KPS, Incheon, Republic of Korea) and fractionated by sieving through mesh screens of different sizes (60, 80, 100, 120, and 160 mesh) to obtain samples with distinct particle-size distributions.

### 2.3. Characterization of Baegjinju Rice Flour

#### 2.3.1. Particle-Size Distribution

The particle-size characteristics of each rice flour fraction were analyzed in powder mode using a particle-size analyzer (LS 13 320, Beckman Coulter, Brea, CA, USA) [13]. The mean particle size and particle-size distribution were recorded for each sample.

#### 2.3.2. Moisture Content of Baegjinju Rice Flour

Following the AOAC method [14], a 1 g portion of each rice flour sample was heated at 105 °C for 24 h in a forced-convection oven (OF4-15SW, Jeio Tech, Daejeon, Republic of Korea) until a constant mass was obtained.

#### 2.3.3. Water-Holding Capacity (WHC)

Water-holding capacity (WHC) was measured by centrifugation [15]: 1 g of rice flour was mixed with 20 mL of distilled water in a conical tube and vortexed for 30 s using a vortex mixer (Vortex-Genie 2, Scientific Industries, Inc., Bohemia, NY, USA). The mixture was incubated in a shaking incubator (ThermoStable IS-20R, DAIHAN Scientific Co., Ltd., Wonju, Republic of Korea) at 30 °C and 100 rpm for 25 min and subsequently centrifuged at 5000× *g* for 10 min using a refrigerated centrifuge (1248R, Gyrozen Co., Ltd., Daejeon, Republic of Korea). After carefully removing the supernatant, WHC was calculated using the following equation:

$$WHC\left(g/g\right)=[(wet\;sediment\;weight-initial\;dry\;sample\;weight)/(initial\;dry\;sample\;weight)].$$

#### 2.3.4. Amylose Content

Amylose content was determined using a colorimetric iodine-binding method [16]. A calibration curve was prepared using potato amylose (A0512-1G, Sigma-Aldrich Chemical Co., St. Louis, MO, USA). Briefly, 40 mg of amylose was combined with 1 mL of 95% ethanol and 9 mL of 1 N NaOH, heated in a water bath (JSIB-22T, JS Research Inc., Gongju, Republic of Korea) at 100 °C for 10 min, and then diluted to 100 mL with distilled water. Rice flour samples weighing 100 mg were subjected to the same dissolution procedure. A 5 mL aliquot of each standard or sample solution was transferred to a volumetric flask, followed by the addition of 1 mL of 1 N acetic acid and 2 mL of iodine–potassium iodide solution. Each mixture was brought to a final volume of 100 mL with distilled water, mixed thoroughly, and allowed to react for 20 min. Absorbance was measured at 620 nm using a UV/Visible spectrophotometer (X-ma 3000 Series, Human Corporation, Seoul, Republic of Korea), and amylose content was calculated based on the standard curve.

#### 2.3.5. Damaged Starch

Damaged starch content was determined using a commercial starch damage assay kit (KSDAM, Megazyme International, Wicklow, Ireland). The assay was conducted in accordance with AACC International Method 76-31.01 [17].

#### 2.3.6. Color of Rice Flour

The L*, a*, and b* values of rice flour samples were measured using a colorimeter (CR-400, Konica Minolta Inc., Tokyo, Japan) [18]. Before analysis, the instrument was calibrated using a white standard plate with values of Y = 86.3, x = 0.3161, and y = 0.3216.

#### 2.3.7. Pasting Properties

The pasting characteristics of the rice flour samples were determined using a Rapid Visco Analyzer (RVA 4500; Perten, Macquarie Park, NSW, Australia) [19]. Rice flour (3 g) and distilled water (25 g) were placed in an RVA canister and mixed. The temperature program consisted of holding the sample at 50 °C for 1 min, heating to and maintaining it at 95 °C for 2 min 30 s, and subsequently cooling it to 50 °C for 1 min 30 s. Peak viscosity, trough viscosity, breakdown viscosity, final viscosity, setback viscosity, and pasting temperature were obtained from the resulting pasting profile.

#### 2.3.8. Textural Properties of Baegjinju Rice Flour Gels

Rice flour gels containing 15% flour (*w/w*) were prepared for texture profile analysis (TPA). A dispersion containing 10 g of rice flour was stirred at 35 °C for 1 min and then heated at 95 °C for 30 min. After heating, the gels were cooled and stored at 4 °C overnight. Textural properties were measured using a texture analyzer (CT3, Brookfield, Middleboro, MA, USA) equipped with a P/5 probe, following the method described in [20]. The test speed was set at 1 mm/s, the compression distance was 10 mm, and the interval between compression cycles was 5 s.

### 2.4. Preparation and Quality Evaluation of Batter-Coated Fried Chicken

#### 2.4.1. Preparation of Fried Chicken Samples

The wheat flour reference batter was prepared using 100 g of wheat flour, 3 g of sodium chloride, 1 g of sodium bicarbonate, and 140 g of distilled water, based on a previously reported wheat flour-based frying batter formulation [21]. The wheat flour amount was standardized to 100 g, while the amounts of the other ingredients were retained. When the same flour-to-water ratio was applied to Baegjinju rice flour, a homogeneous batter was not formed, and visible dry agglomerates remained in the mixture. Preliminary formulation trials were therefore conducted by gradually increasing the amount of water added to 100 g of rice flour. A water level of 180 g was selected as the minimum amount required to obtain a homogeneous batter without visible dry agglomerates and to form a continuous coating on the chicken surface. Accordingly, each rice flour batter was prepared using 100 g of the designated rice flour fraction, the same amounts of sodium chloride (3 g) and sodium bicarbonate (1 g), and 180 g of distilled water. The same water level was applied to all rice flour treatments to maintain particle size as the principal formulation variable. Each batter was manually stirred continuously for 5 min using a laboratory spatula until a homogeneous mixture was obtained.

Refrigerated chicken breast purchased 1 day before experimentation was used to maintain freshness. The meat was cut into pieces measuring 3 cm × 3 cm × 2 cm in width, length, and height, respectively, to minimize variation among samples [22]. Chicken pieces were completely immersed in the designated batter and allowed to drain briefly after removal to eliminate excess batter. The coated samples were then fried at 170 °C for 3 min 30 s using a deep fryer (DKR-113, Delki Co., Ltd., Goyang, Republic of Korea). The fried samples were allowed to cool at room temperature until they reached 40 °C before physicochemical analyses and texture analyses were performed.

#### 2.4.2. Pick-Up and Cooking Loss

Batter pick-up was determined from the increase in sample weight after battering, whereas cooking loss was calculated from the change in sample weight during frying [1].

$$\mathrm{Pick-up}\;(\%)=\frac{(B-I)}{I}\times 100,$$

(1) $$\mathrm{Cooking}\;\mathrm{loss}\;(\%)=\frac{\left(B-F\right)}{I}\times 100,$$

where I is the weight of the raw chicken sample before batter coating, B is the weight of the same sample immediately after batter coating and before frying, and F is the weight of the fried sample after cooling to 40 °C.

#### 2.4.3. Whole-Product Moisture Content of Batter-Coated Fried Chicken

The whole-product moisture content of the batter-coated fried chicken was determined using a gravimetric oven-drying procedure. Each intact fried chicken piece, including both the batter coating and the meat core, was weighed and dried in a forced-convection oven (OF4-15SW, Jeio Tech, Daejeon, Republic of Korea) at 105 °C. The samples were periodically weighed during drying, and drying was continued until a constant mass was obtained. Constant mass was achieved within 48 h for all samples. After drying, the samples were equilibrated in a desiccator for 1 h before the final weight was recorded. The resulting values represent the moisture content of the entire batter-coated fried product, including both the coating and the meat core.

#### 2.4.4. Texture Profile Analysis of Batter-Coated Fried Chicken

Texture profile analysis of the fried chicken samples was conducted using a texture analyzer (CT3, Brookfield, Middleboro, MA, USA) fitted with a TA4/1000 cylindrical probe with a diameter of 38.1 mm [22]. The test conditions were 5 mm/s speed, 10 mm compression, 10 g trigger load, and 5 s interval. These parameters allowed for the determination of hardness, cohesiveness, springiness, adhesiveness, and chewiness.

### 2.5. Statistical Analysis

Statistical analyses were carried out using SPSS software version 26 (SPSS Inc., Chicago, IL, USA). Unless otherwise specified, measurements were performed in triplicate, and the data were expressed as mean ± standard deviation. Differences among treatments were evaluated using one-way analysis of variance (ANOVA). Duncan’s multiple range test was applied for mean comparison at a significance level of *p* < 0.05. Pearson’s correlation analysis was performed to evaluate the relationship between WHC and peak viscosity.

## 3. Results and Discussion

### 3.1. Characteristics of Baegjinju Rice Flour

#### 3.1.1. Particle-Size Distribution of Baegjinju Rice Flour

Baegjinju rice flour particle-size distribution showed a clear and systematic decrease with increasing mesh size (Table 1). The mean particle size decreased significantly from 176.95 ± 3.60 µm (60-mesh) to 59.87 ± 0.30 µm in the 160-mesh fraction (*p* < 0.05). Corresponding reductions occurred for D10, D50, and D90 values, indicating that dry milling and sieving effectively separated rice flour into distinct fractions with progressively finer distributions.

Specifically, D10 values decreased from 52.62 ± 0.41 µm to 8.43 ± 0.04 µm, D50 values from 147.45 ± 0.49 µm to 44.14 ± 0.38 µm, and D90 values from 314.30 ± 4.53 µm to 139.85 ± 0.21 µm as mesh size increased. The pronounced D90 reduction indicates efficient removal of coarse particles, resulting in improved homogeneity.

Particle-size reduction increases specific surface area and modifies particle packing, affecting hydration capacity, starch gelatinization, and viscosity development in subsequent analyses. The well-controlled fractionation achieved provides a robust foundation for interpreting particle-size-dependent differences in batter performance and fried product quality.

#### 3.1.2. Physicochemical Properties

Baegjinju rice flour physicochemical properties were significantly affected by particle size, as evidenced by changes in moisture content, WHC, amylose content, damaged starch content, and color attributes (Table 2). These parameters collectively influence hydration behavior and functional performance in batter systems.

Moisture content decreased progressively with decreasing particle size, from 12.26 ± 0.41% (60-mesh) to 8.76 ± 0.46% (160-mesh). This reduction is attributable to increased surface area of finer particles, which promotes moisture loss during milling and handling [6]. Lower inherent moisture levels in finer fractions may in turn influence water uptake during batter preparation.

Conversely, WHC increased significantly as particle size decreased, ranging from 1.34 ± 0.01 g/g to 1.84 ± 0.02 g/g. Enhanced WHC in finer fractions is associated with increased surface area, higher porosity, and greater exposure of hydrophilic groups capable of binding water [15,23]. High WHC critically affects water distribution, viscosity development, and batter matrix formation because it directly influences these key functional properties.

Amylose content exhibited a gradual increase with decreasing particle size, increasing from 13.76 ± 0.34% to 16.02 ± 0.06%, explained by increased accessibility or partial amylose release from granules during fine milling [24]. Amylose plays a key role in gel formation through hydrogen bonding, contributing to viscosity development and internal network structure in hydrated starch systems [16].

Damaged starch content increased markedly with decreasing particle size, from 7.18 ± 0.14% (60-mesh) to 17.98 ± 0.11% (160-mesh), reflecting intensified mechanical stress during milling. Although increased damaged starch enhances water binding and influences viscoelastic behavior, excessive damage may impair gelatinization stability and accelerate retrogradation [6,8]. Therefore, an appropriate balance between improved hydration and structural stability is essential when evaluating finely milled rice flours [25]. Notably, the 160-mesh fraction, despite having the highest damaged starch content (17.98%), still showed higher WHC and batter pick-up, lower cooking loss, and greater moisture retention. This suggests that, within the range examined, the beneficial effects of enhanced hydration and coating formation remained dominant even in the finest fraction.

Color attributes were also influenced by particle size. Lightness (L*) increased with decreasing particle size, whereas yellowness (b*) decreased, attributable to enhanced light scattering by smaller particles and dilution or redistribution of colored components in finer fractions [15].

Overall, particle-size reduction induced coordinated changes in hydration properties, starch composition, and optical characteristics, underscoring the importance of particle-size control in optimizing functional performance in batter applications.

#### 3.1.3. Pasting Properties

**Figure 1:**
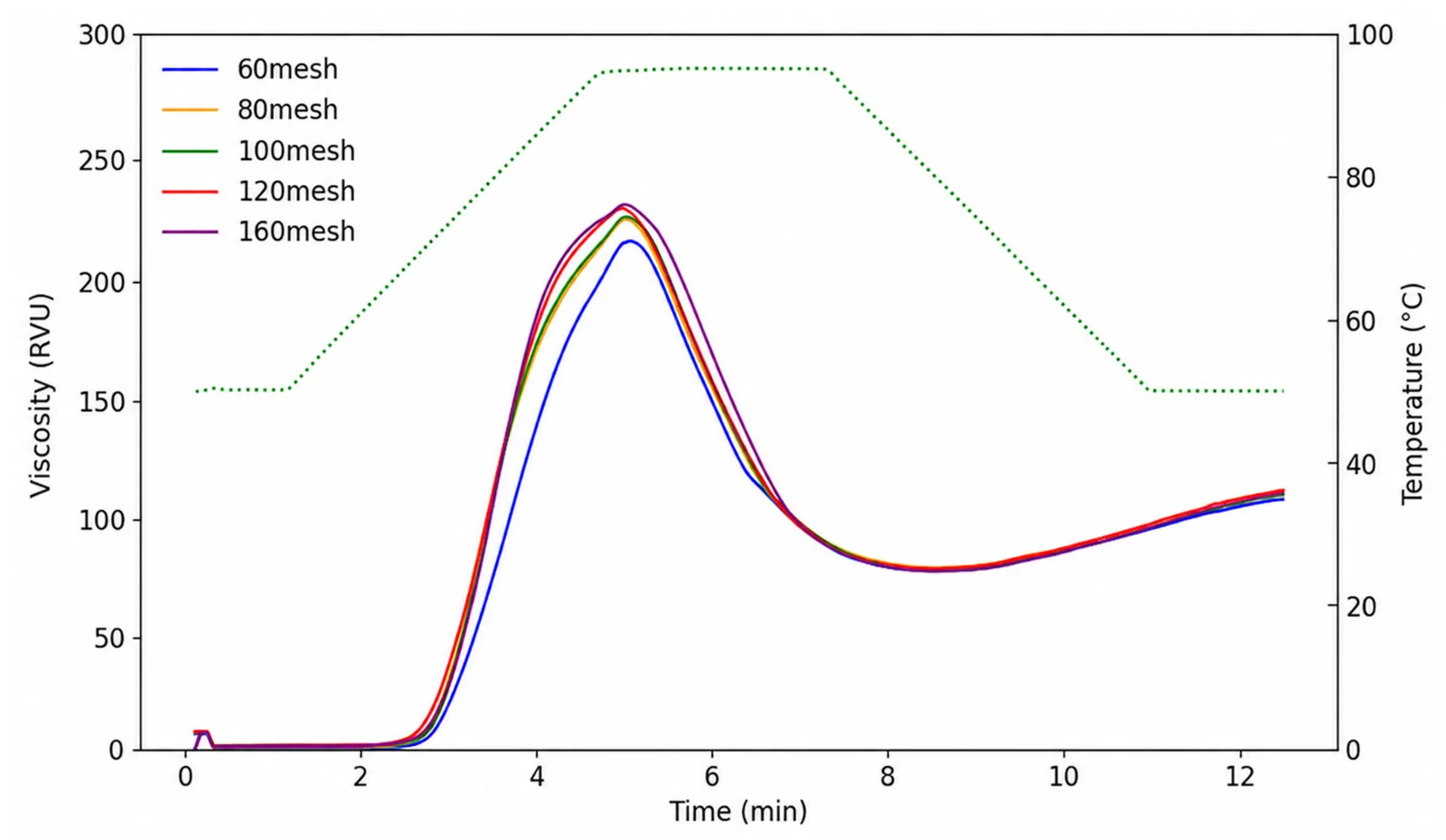
Representative RVA pasting curves of Baegjinju rice flour fractions with different particle sizes (60-, 80-, 100-, 120-, and 160-mesh sieves). The dashed line indicates the temperature profile during the heating–cooling cycle. Quantitative viscosity parameters and statistical comparisons are presented in Table 3.

The pasting properties of Baegjinju rice flour fractions were significantly influenced by particle size (*p* < 0.05) (Table 3). The particle-size-dependent differences in viscosity development are also illustrated by the representative RVA pasting curves shown in Figure 1. Changes in viscosity parameters and pasting temperature indicate that particle size critically regulates starch gelatinization and viscosity development. Peak viscosity increased progressively with decreasing particle size, from 224.44 ± 1.05 RVU (60-mesh) to 239.50 ± 0.38 RVU (160-mesh), suggesting enhanced hydration and swelling capacity in finer particles [26]. The strong correlation between WHC and peak viscosity (r = 0.99, *p* < 0.01) supports this relationship. Breakdown viscosity also increased from 141.94 ± 1.09 RVU to 166.39 ± 0.76 RVU with decreasing particle size, indicating greater susceptibility of the swollen starch paste to thermal and shear disruption, possibly in association with enhanced granule swelling and increased starch damage in the finer fractions.

Setback viscosity, calculated as final viscosity minus trough viscosity, showed an overall increasing tendency with decreasing particle size, ranging from 31.11 ± 0.38 RVU in the 60-mesh fraction to 33.58 ± 0.58 RVU in the 160-mesh fraction. Although apparent amylose content increased with decreasing particle size, setback viscosity did not increase progressively. This indicates that cooling-stage viscosity recovery was likely influenced by combined changes in amylose content, damaged starch, and hydration behavior rather than by amylose content alone.

Pasting temperature decreased from 73.62 °C to 70.43 °C with decreasing particle size, indicating that gelatinization occurred more readily in smaller particles. This reduction is attributed to increased granule disruption, higher damaged starch levels, and enhanced water accessibility from fine milling. These changes suggest that particle-size reduction facilitates earlier viscosity development during heating, which is an important functional advantage for batter systems exposed to rapid thermal processing.

Overall, these results indicate that particle-size reduction systematically modifies pasting behavior by enhancing hydration, promoting viscosity development, and reducing gelatinization temperature. These effects underscore the importance of particle-size control in optimizing the functional performance.

#### 3.1.4. Textural Properties of Baegjinju Rice Flour Gels

TPA showed that textural properties of rice flour gels prepared with Baegjinju rice flour were significantly affected by particle size (Table 4). Significant differences in hardness, adhesiveness, cohesiveness, springiness, and gumminess confirm that particle size critically determines mechanical behavior.

Gel hardness increased as mesh size increased (i.e., as particle size decreased), with the lowest value in the 60-mesh sample (22.33 ± 1.15 g) and highest in the 160-mesh sample (31.00 ± 2.00 g). This trend suggests finer particles contributed to gel matrix formation with greater deformation resistance. The increased hardness in finer fractions is associated with enhanced hydration, increased damaged starch content, and tighter particle packing, which collectively promote a more compact and mechanically stable network [27].

Adhesiveness increased significantly with increasing mesh size, ranging from 0.90 ± 0.10 mJ in the 60-mesh sample to 1.81 ± 0.36 mJ in the 160-mesh sample. Higher adhesiveness in finer fractions reflects improved binding capacity within the gel matrix, attributable to increased surface area and greater availability of hydrophilic sites for water interaction, enhancing interparticle bonding and cohesion [28].

Cohesiveness increased as particle size decreased, with values increasing from 0.35 ± 0.03 to 0.58 ± 0.11. Increased cohesiveness indicates more integrated internal structure, suggesting finer particles facilitate more continuous and stable gel network formation. This is consistent with previous studies reporting that reduced particle size enhances structural integrity [24].

Springiness exhibited marked increase with higher mesh sizes, progressing from 6.90 ± 0.16 (60-mesh) to 12.12 ± 0.48 mm (160-mesh). This increase indicates finer particles contributed to more elastic gel structure formation, likely owing to improved water retention and flexible starch gel network development during hydration and gelatinization [29].

Gumminess increased as particle size decreased, ranging from 12.67 ± 1.15 g to 17.67 ± 1.53 g. Higher gumminess in finer fractions suggests denser, more compact gel structure formation with greater deformation resistance. Similar particle-size-dependent changes in textural properties have been reported in flour-based systems [30].

Overall, these results demonstrate that decreasing particle size increased the hardness, adhesiveness, cohesiveness, springiness, and gumminess of Baegjinju rice flour gels, whereas coarser fractions produced softer and less integrated gel matrices. These findings demonstrate that particle size substantially influences the textural and structural properties of Baegjinju rice flour gels.

### 3.2. Quality Characteristics of Batter-Coated Fried Chicken

#### 3.2.1. Physical Properties of Batter-Coated Fried Chicken

**Figure 2:**
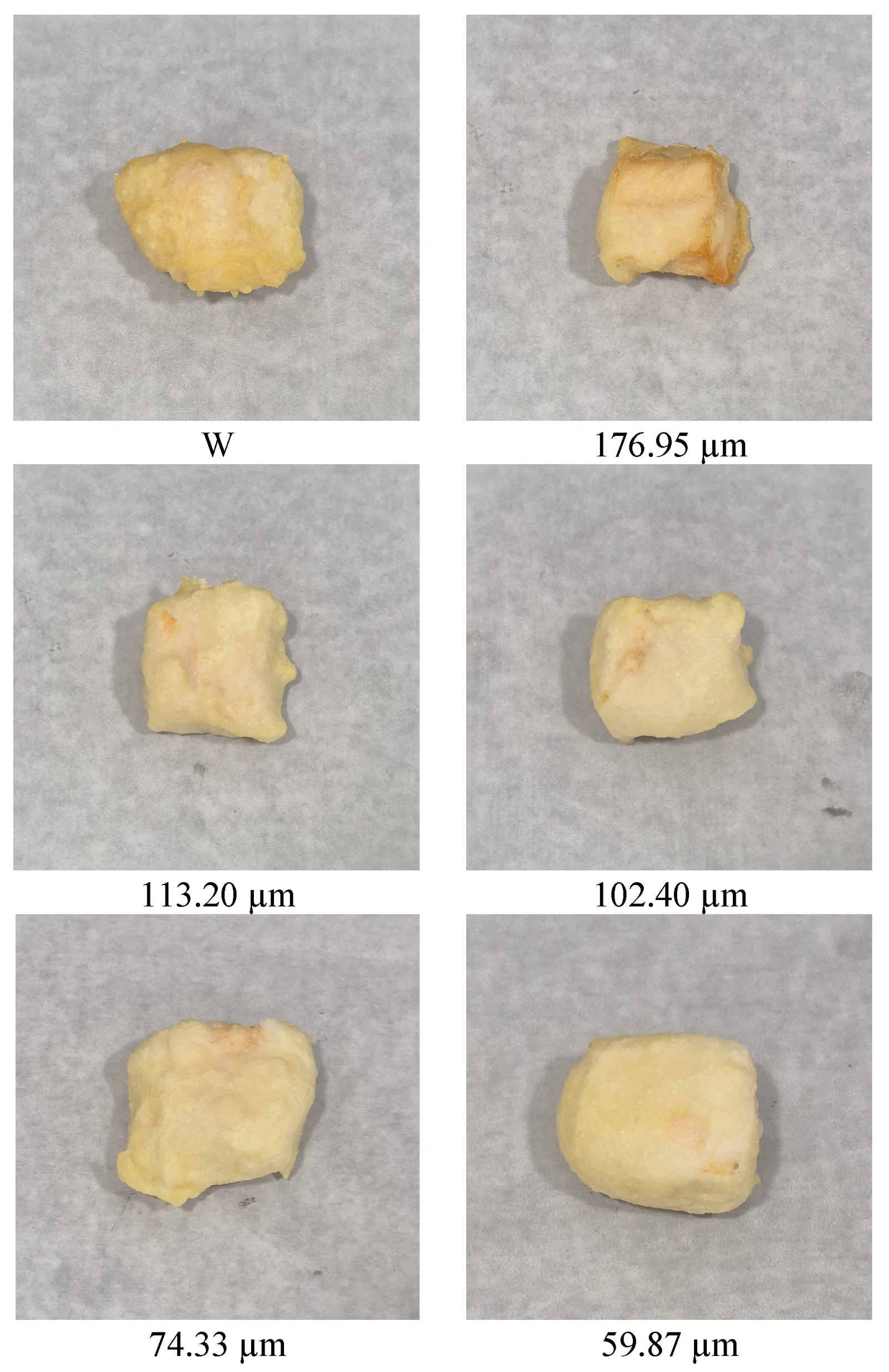
Representative images of batter-coated deep-fat-fried chicken prepared with the conventional wheat flour reference batter (W) and Baegjinju rice flour fractions, with the mean particle sizes indicated in each panel.

Physical properties of fried chicken, including batter pick-up, cooking loss, and moisture content, were significantly influenced by the particle size of the Baegjinju rice flour used in the batter formulation (Table 5). These parameters reflect coating behavior during frying and provide insight into the batter system’s ability to adhere to substrate, retain moisture, and maintain structural integrity during thermal processing. Representative images of the batter-coated fried chicken samples prepared with the different Baegjinju rice flour particle-size fractions and the wheat flour reference are shown in Figure 2.

Batter pick-up increased markedly with decreasing particle size. The lowest pick-up occurred in the 60-mesh sample (7.54 ± 2.97%), whereas the highest occurred in the 160-mesh sample (36.68 ± 4.81%). This pronounced increase indicates that finer particles increased the amount of batter adhering to the chicken surface, likely owing to increased surface contact and the enhanced hydration characteristics of the finer rice flour fractions [6]. Wheat flour batter pick-up (17.23 ± 1.34%) was comparable to that of the 100-mesh rice flour batter, suggesting appropriate particle-size adjustment enables rice flour batters to achieve adhesion performance like wheat flour. The increase in batter pick-up with decreasing particle size was consistent with previous findings that changes in batter formulation and viscosity can influence coating pick-up in battered chicken products [3]. Similar differences in wet pick-up among batters prepared from different rice cultivars have also been reported, indicating that rice flour characteristics can influence batter pick-up [11].

Cooking loss decreased progressively as rice flour particle size decreased. The highest cooking loss occurred in the 60-mesh sample (41.82 ± 0.81%), whereas the lowest occurred in the 160-mesh sample (16.40 ± 2.70%). The reduced cooking loss in the finer fractions suggests that these batters formed more continuous and cohesive coatings that were more effective in limiting moisture evaporation during frying [1,7]. Notably, 160-mesh rice flour batter cooking loss (16.40 ± 2.70%) was comparable to that of wheat flour batter (16.34 ± 0.25%), showing a similar cooking-loss response under the formulations used in this study. A recent study on gluten-free crispy chicken similarly showed that coating formulation affected cooking loss [4]. Although the formulation and processing conditions differed from those of the present study, this finding supports the present observation that batter characteristics play an important role in controlling moisture loss during frying.

Whole-product moisture content of the batter-coated fried chicken increased significantly with decreasing rice flour particle size. The 60-mesh sample exhibited the lowest whole-product moisture content (48.22 ± 1.94%), whereas the 160-mesh sample showed the highest value (62.21 ± 1.55%). Because intact fried chicken pieces containing both the coating and the meat core were analyzed, these values represent the overall moisture content of the final fried product and should not be interpreted as the moisture content of the meat component alone. The higher whole-product moisture content observed in samples coated with finer rice flour fractions may reflect differences in coating characteristics and overall water retention during frying. The whole-product moisture content of the 160-mesh sample was comparable to that of the wheat flour reference (61.46 ± 2.38%).

Previous studies have shown that rice-flour-based batters can reduce oil absorption compared with wheat-flour-based systems and that oil uptake is influenced by starch composition and coating characteristics [7]. In the present study, particle-size reduction markedly altered batter pick-up, cooking loss, and moisture retention, suggesting changes in coating structure and mass transfer that could also affect oil absorption. However, oil content was not directly measured; therefore, the effect of Baegjinju rice flour particle size on oil uptake cannot be determined from the present results.

#### 3.2.2. Textural Properties of Batter-Coated Fried Chicken

TPA showed that textural characteristics of fried chicken coated with Baegjinju rice flour batter were affected by particle size (Table 6). Significant differences in hardness, adhesiveness, cohesiveness, springiness, and chewiness confirm that particle size critically determined the mechanical behavior of the fried coating.

Hardness decreased progressively with decreasing particle size. The highest hardness occurred in the 60-mesh sample (4500.67 ± 367.85 g), whereas the lowest occurred in the 160-mesh sample (1598.67 ± 588.11 g). This marked reduction indicates batters formulated with finer particles formed a less rigid crust structure after frying. Softer texture associated with finer particles may be related to differences in coating characteristics and the higher whole-product moisture content observed in the finer rice flour treatments [31]. Importantly, the hardness of the wheat flour reference sample (1490.75 ± 300.68 g) was within the range for finer rice flour fractions, indicating comparable textural performance between wheat flour and finely milled Baegjinju rice flour.

Adhesiveness increased significantly with decreasing particle size, from 0.93 ± 0.99 mJ (60-mesh) to 3.43 ± 1.06 mJ (160-mesh). Increased adhesiveness reflects enhanced interfacial binding between the batter and chicken surface, explained by the greater specific surface area and improved hydration capacity of finer particles. These characteristics promote stronger adhesive interactions and contribute to more uniform and stable coating formation during frying [28,31]. The adhesiveness of the 80- and 100-mesh rice-flour-coated samples was comparable to that of the wheat flour reference (1.65 ± 0.34 mJ). Cohesiveness increased progressively with decreasing particle size, from 0.45 ± 0.03 in the 60-mesh sample to 0.63 ± 0.02 in the 160-mesh sample. The cohesiveness of the 160-mesh rice-flour-coated sample was comparable to that of the wheat flour reference (0.60 ± 0.03), suggesting that the finer rice flour fraction maintained a cohesive fried coating structure under the conditions used in this study.

Springiness decreased at lower particle sizes, with the highest value in the 60-mesh sample (7.98 ± 0.36 mm) and the lowest in the 160-mesh sample (6.39 ± 0.47 mm). This pattern suggests coarser particles produced more elastic and resilient crust structure, potentially owing to more pronounced starch-based framework formation within batter matrix. The springiness of the wheat flour reference (7.75 ± 0.29 mm) was similar to that of the 60- and 80-mesh rice-flour-coated samples.

Chewiness followed a similar trend to hardness, decreasing significantly with decreasing particle size. The highest chewiness occurred in the 60-mesh sample (192.13 ± 8.19 g), whereas the lowest occurred in the 160-mesh sample (63.13 ± 29.87 g). Wheat-flour-coated sample chewiness (70.87 ± 13.02 g) was closer to that of 120- and 160-mesh rice flour batters, indicating finer fractions produced textural characteristics comparable to conventional wheat flour coatings. Chewiness reduction with decreasing particle size is likely driven by increased moisture retention and reduced crust density, resulting in a more tender and less resistant fried product [8].

Previous studies have likewise shown that changes in flour type and coating formulation markedly influence the instrumental texture of battered chicken products [3]. More recently, Ates Cakiroglu et al. [4] reported substantial changes in hardness and other TPA parameters after cooking gluten-free coated chicken, emphasizing the strong dependence of final product texture on coating composition and processing conditions.

## 4. Conclusions

This study systematically evaluated how the particle size of Baegjinju rice flour affected its functional properties and the quality characteristics of batter-coated fried chicken. Reducing particle size from 176.95 to 59.87 µm increased rice flour gel cohesiveness from 0.35 to 0.58 and adhesiveness from 0.90 to 1.81 mJ. In the resulting fried chicken, hardness decreased from 4500.67 to 1598.67 g and chewiness decreased from 192.13 to 63.13 g, accompanied by a higher whole-product moisture content and a less rigid fried coating. Particle-size reduction also increased batter pick-up from 7.54 to 36.68% and whole-product moisture content of the fried chicken from 48.22 to 62.21%, while cooking loss decreased from 41.82 to 16.40%. Although damaged starch content increased from 7.18 to 17.98% with decreasing particle size, the beneficial effects associated with enhanced hydration and coating performance remained evident despite the increase in damaged starch content. Consequently, the 120- and 160-mesh fractions, with mean particle sizes of 74.33 and 59.87 µm, respectively, produced fried chicken with hardness and chewiness comparable to those of the wheat flour reference.

A limitation of this study is that different water levels were required to produce workable wheat and rice flour batters. Accordingly, the effects of flour type and formulation water could not be fully separated in comparisons with the wheat flour reference. Nevertheless, all Baegjinju rice flour treatments were prepared under identical formulation conditions, allowing particle-size-dependent differences among the rice flour fractions to be evaluated under controlled conditions. Under the formulation and frying conditions used in this study, Baegjinju rice flour with mean particle sizes of approximately 59.87–74.33 µm, corresponding to the 120–160-mesh fractions, may represent a practical particle-size range for fried chicken batter applications. Overall, these findings demonstrate that particle-size control can enhance the applicability of Baegjinju rice flour in deep-fat-frying systems and provide a scientific basis for the development of rice-flour-based fried products.

## Figures and Tables

**Table 1:** Particle-size distribution of Baegjinju rice flour fractions obtained using different sieve mesh sizes.

Sieve Designation (mesh)	Particle-Size Distributions			
	Mean (µm)	D10 (µm)	D50 (µm)	D90 (µm)
60	176.95 ± 3.60 ^a^	52.62 ± 0.41 ^a^	147.45 ± 0.49 ^a^	314.30 ± 4.53 ^a^
80	113.20 ± 0.14 ^b^	18.30 ± 0.74 ^b^	86.26 ± 1.60 ^b^	213.45 ± 0.35 ^b^
100	102.40 ± 0.71 ^c^	15.82 ± 0.24 ^c^	79.34 ± 1.23 ^c^	190.70 ± 0.28 ^c^
120	74.33 ± 0.42 ^d^	9.13 ± 0.03 ^d^	51.88 ± 0.53 ^d^	164.85 ± 1.48 ^d^
160	59.87 ± 0.30 ^e^	8.43 ± 0.04 ^d^	44.14 ± 0.38 ^e^	139.85 ± 0.21 ^e^

Values are the mean ± SD (*n* = 3). Different letters (a–e) in a column indicate significant differences (*p* < 0.05) according to Duncan’s test.

**Table 2:** Physicochemical properties of rice flours with different particle sizes.

Size (mesh)	Physicochemical Properties				Color		
	Moisture Content (%)	Water Holding Capacity (g/g)	Amylose Content (%)	Damaged Starch (%)	*L* *	a*	b*
60	12.26 ± 0.41 ^a^	1.34 ± 0.01 ^e^	13.76 ± 0.34 ^e^	7.18 ± 0.14 ^e^	92.66 ± 0.05 ^e^	−0.21 ± 0.04 ^b^	5.47 ± 0.03 ^a^
80	11.09 ± 0.36 ^b^	1.45 ± 0.02 ^d^	14.43 ± 0.33 ^d^	10.32 ± 0.22 ^d^	93.45 ± 0.12 ^d^	−0.18 ± 0.07 ^ab^	4.41 ± 0.04 ^b^
100	10.56 ± 0.18 ^bc^	1.58 ± 0.11 ^c^	15.05 ± 0.47 ^c^	11.80 ± 0.03 ^c^	93.86 ± 0.05 ^c^	−0.15 ± 0.03 ^ab^	4.33 ± 0.04 ^c^
120	9.88 ± 0.55 ^c^	1.71 ± 0.01 ^b^	15.48 ± 0.18 ^b^	16.86 ± 0.02 ^b^	94.10 ± 0.17 ^b^	−0.14 ± 0.01 ^ab^	3.70 ± 0.06 ^d^
160	8.76 ± 0.46 ^d^	1.84 ± 0.02 ^a^	16.02 ± 0.06 ^a^	17.98 ± 0.11 ^a^	94.40 ± 0.62 ^a^	−0.12 ± 0.02 ^a^	3.92 ± 0.05 ^e^

The values represent Baegjinju rice flour fractions obtained using 60-, 80-, 100-, 120-, and 160-mesh sieves. Values are the mean ± SD (*n* = 3). Different letters (a–e) in a column indicate significant differences (*p* < 0.05) according to Duncan’s test.

**Table 3:** Rapid visco analysis of viscosity and pasting temperature for Baegjinju rice flour.

Size (mesh)	Viscosity (RVU)					Pasting Temp (°C)
	Peak	Trough	Break Down	Final	Setback	
60	224.44 ± 1.05 ^e^	82.50 ± 1.44 ^a^	141.94 ± 1.09 ^e^	113.61 ± 0.51 ^a^	31.11 ± 0.38 ^c^	73.62 ± 0.49 ^a^
80	229.47 ± 0.05 ^d^	78.05 ± 0.68 ^b^	151.42 ± 0.63 ^d^	110.86 ± 0.79 ^b^	32.81 ± 0.27 ^b^	72.35 ± 0.10 ^b^
100	232.97 ± 0.53 ^c^	77.20 ± 2.29 ^b^	155.78 ± 2.76 ^c^	109.92 ± 2.29 ^b^	32.72 ± 0.05 ^b^	72.30 ± 0.05 ^b^
120	236.50 ± 1.15 ^b^	73.72 ± 0.24 ^c^	162.78 ± 1.40 ^b^	107.11 ± 0.77 ^c^	33.39 ± 0.53 ^ab^	70.72 ± 0.03 ^c^
160	239.50 ± 0.38 ^a^	73.11 ± 0.68 ^c^	166.39 ± 0.76 ^a^	106.69 ± 0.10 ^c^	33.58 ± 0.58 ^a^	70.43 ± 0.38 ^c^

Values are the mean ± SD (*n* = 3). Different letters (a–e) in a column indicate significant differences (*p* < 0.05) according to Duncan’s test.

**Table 4:** Texture profile analysis (TPA) of rice flour gel prepared with different rice flour particle sizes.

Size (mesh)	Hardness (g)	Adhesiveness (mJ)	Cohesiveness	Springiness (mm)	Gumminess (g)
60	22.33 ± 1.15 ^d^	0.90 ± 0.10 ^d^	0.35 ± 0.03 ^d^	6.90 ± 0.16 ^d^	12.67 ± 1.15 ^d^
80	24.33 ± 0.58 ^cd^	1.28 ± 0.05 ^c^	0.42 ± 0.02 ^cd^	7.73 ± 0.11 ^c^	14.00 ± 1.00 ^cd^
100	25.33 ± 0.58 ^c^	1.40 ± 0.08 ^bc^	0.46 ± 0.02 ^bc^	8.38 ± 0.05 ^c^	15.00 ± 1.00 ^bc^
120	27.67 ± 1.15 ^b^	1.61 ± 0.11 ^ab^	0.53 ± 0.03 ^ab^	9.51 ± 0.19 ^b^	16.33 ± 1.15 ^ab^
160	31.00 ± 2.00 ^a^	1.81 ± 0.36 ^a^	0.58 ± 0.11 ^a^	12.12 ± 0.48 ^a^	17.67 ± 1.53 ^a^

Values are the mean ± SD (*n* = 3). Different letters (a–d) in a column indicate significant differences (*p* < 0.05) according to Duncan’s test.

**Table 5:** Physical properties of batter-coated fried chicken prepared with different particle sizes of rice flour.

Size (mesh)	Physical Properties		
	Pick Up (%)	Cooking Loss (%)	Whole-Product Moisture Content (%)
60	7.54 ± 2.97 ^d^	41.82 ± 0.81 ^a^	48.22 ± 1.94 ^d^
80	12.84 ± 0.13 ^cd^	34.82 ± 2.42 ^b^	52.42 ± 2.60 ^c^
100	16.91 ± 1.99 ^c^	29.58 ± 0.90 ^c^	57.55 ± 3.73 ^b^
120	27.71 ± 6.91 ^b^	25.82 ± 1.20 ^d^	59.19 ± 1.35 ^ab^
160	36.68 ± 4.81 ^a^	16.40 ± 2.70 ^e^	62.21 ± 1.55 ^a^
W	17.23 ± 1.34 ^c^	16.34 ± 0.25 ^e^	61.46 ± 2.38 ^ab^

W represents the conventional wheat flour reference batter prepared with 140 g of water, whereas the Baegjinju rice flour batters were prepared with 180 g of water, as described in Section 2.4.1. Whole-product moisture content was determined using intact batter-coated fried chicken pieces, including both the coating and the meat core. Values are the mean ± SD (*n* = 3). Different letters (a–e) in a column indicate significant differences (*p* < 0.05) according to Duncan’s test.

**Table 6:** Texture profile analysis (TPA) of fried chicken coated with Baegjinju rice flour and wheat flour batters across different particle sizes.

Size (mesh)	Hardness (g)	Adhesiveness (mJ)	Cohesiveness	Springiness (mm)	Chewiness (g)
60	4500.67 ± 367.85 ^a^	0.93 ± 0.99 ^c^	0.45 ± 0.03 ^d^	7.98 ± 0.36 ^a^	192.13 ± 8.19 ^a^
80	4284.33 ± 708.11 ^a^	1.72 ± 0.74 ^bc^	0.49 ± 0.03 ^cd^	7.58 ± 0.41 ^a^	160.93 ± 15.68 ^b^
100	3368.67 ± 250.44 ^ab^	2.07 ± 0.93 ^bc^	0.52 ± 0.02 ^bc^	7.39 ± 0.29 ^ab^	117.63 ± 10.52 ^c^
120	2212.75 ± 1097.38 ^c^	2.70 ± 1.22 ^ab^	0.53 ± 0.02 ^b^	6.95 ± 0.42 ^bc^	72.77 ± 20.56 ^d^
160	1598.67 ± 588.11 ^c^	3.43 ± 1.06 ^a^	0.63 ± 0.02 ^a^	6.39 ± 0.47 ^c^	63.13 ± 29.87 ^d^
W	1490.75 ± 300.68 ^c^	1.65 ± 0.34 ^bc^	0.60 ± 0.03 ^a^	7.75 ± 0.29 ^a^	70.87 ± 13.02 ^d^

W represents the conventional wheat flour reference batter prepared with 140 g of water, whereas the Baegjinju rice flour batters were prepared with 180 g of water, as described in Section 2.4.1. Values are the mean ± SD (*n* = 3). Different letters (a–d) in a column indicate significant differences (*p* < 0.05) according to Duncan’s test.

## Data Availability

The original contributions presented in this study are included in the article. Further inquiries can be directed to the corresponding author.

## Abbreviations

The following abbreviations are used in this manuscript:

WHC	Water-holding capacity
TPA	Texture profile analysis
ANOVA	Analysis of variance
SD	Standard deviation
